# Fracture and Embedment Behavior of Brittle Submicrometer Spherical Particles Fabricated by Pulsed Laser Melting in Liquid Using a Scanning Electron Microscope Nanoindenter

**DOI:** 10.3390/nano11092201

**Published:** 2021-08-26

**Authors:** Daizen Nakamura, Naoto Koshizaki, Nobuyuki Shishido, Shoji Kamiya, Yoshie Ishikawa

**Affiliations:** 1 Graduate School of Engineering, Hokkaido University, Sapporo 060-8628, Japan; nakamura.daizen@frontier.hokudai.ac.jp; 2 Faculty of Science and Engineering, Kindai University, Higashiosaka 577-8502, Japan; shishido@mech.kindai.ac.jp; 3 Department of Electrical and Mechanical Engineering, Nagoya Institute of Technology, Nagoya 466-8555, Japan; kamiya.shoji@nitech.ac.jp; 4 Research Institute for Advanced Electronics and Photonics, National Institute of Advanced Industrial Science and Technology (AIST), Tsukuba 305-8565, Japan

**Keywords:** pulsed laser melting in liquid, spherical submicrometer particles, particle fracture, particle embedment, Brinell hardness, titanium carbide

## Abstract

Generally, hard ceramic carbide particles, such as B_4_C and TiC, are angulated, and particle size control below the micrometer scale is difficult owing to their hardness. However, submicrometer particles (SMPs) with spherical shape can be experimentally fabricated, even for hard carbides, via instantaneous pulsed laser heating of raw particles dispersed in a liquid (pulsed laser melting in liquid). The spherical shape of the particles is important for mechanical applications as it can directly transfer the mechanical force without any loss from one side to the other. To evaluate the potential of such particles for mechanical applications, SMPs were compressed on various substrates using a diamond tip in a scanning electron microscope. The mechanical behaviors of SMPs were then examined from the obtained load–displacement curves. Particles were fractured on hard substrates, such as SiC, and fracture strength was estimated to be in the GPa range, which is larger than their corresponding bulk bending strength and is 10–40% of their ideal strength, as calculated using the density-functional theory. Contrarily, particles can be embedded into soft substrates, such as Si and Al, and the local hardness of the substrate can be estimated from the load–displacement curves as a nanoscale Brinell hardness measurement.

## 1. Introduction

Spherical submicrometer particles (SMPs) are larger than well-studied nanoparticles (NPs) but have been an interesting research topic owing to their mechanical applications. The spherical shape of the particles is important, as it can directly transfer the mechanical force without any loss from one side to the other. Submicrometer size is another important factor because suitable fabrication techniques via a top-down approach, such as milling, or a bottom-up approach, such as the nucleation process, are currently unavailable. However, the use of spacers, milling agents, lubricant fillers, etc., has been proposed and is in demand [1,2].

Ductile SMPs made of polymers or glasses are relatively easy to fabricate and are commercially available. Spherical SMPs composed of NP aggregates have also been reported for TiO_2_ [3]. However, these SMPs are polycrystalline or porous, and, therefore, their mechanical properties are not good, owing to the high density of grain boundaries or nanopores, which act as defect sources [4].

Our group reported a new fabrication technique for various spherical SMPs, which is called pulsed laser melting in liquid (PLML) [5,6,7,8,9]. In this process, spherical SMPs are synthesized by applying pulsed laser irradiation with relatively weak laser fluence onto raw colloidal NPs dispersed in a liquid medium. Raw aggregated or agglomerated NPs in a liquid medium are selectively heated and melted via unfocused pulsed laser irradiation to form larger molten droplets due to the temperature increase over the melting point. Subsequently, the particles are quenched with the surrounding liquid to produce crystalline non-porous spherical SMPs [10,11].

PLML appears similar to the well-studied pulsed laser ablation in liquid (PLAL) for NP fabrication [12,13,14], as both are laser processes in liquid medium. However, PLML is not a plasma process like PLAL but a thermal process at high temperatures, ranging from 2000 to 4000 K, due to the difference in the applied laser fluence [15,16]. Therefore, non-porous SMPs of high-temperature materials, such as B, W, TiO_2_, and ZnO, can be fabricated via a simple transient melting process, and B_4_C SPMs can be reactively fabricated via laser melting of raw B particles in ethanol (as a carbon source) [5,6]. Thus, SMPs obtained by PLML are unique in fabrication as well as possible mechanical applications.

However, only a few studies on mechanical properties of hard, brittle, crystalline SMPs have been reported so far [4,17]. In our previous report, brittle non-porous SMPs of B_4_C and TiO_2_ were fabricated via PLML, and their mechanical properties were measured using a nanoindenter equipped in a SEM [4]. In that experiment, the particles were placed on a hard SiC substrate and pressed using a diamond indenter. Their fracture strengths were 40–50% of the ideal strength calculated using the density functional theory (DFT) and somewhat stronger than the bending strength of bulk B_4_C and TiO_2_. Thus, SMPs obtained via PLML were harder compared with those obtained using other techniques, such as chemical methods, and are promising for various mechanical applications.

Here, by extending our previous studies on the mechanical property measurements of SMPs fabricated via PLML [4], indentation tests of various combinations of hard and brittle SMPs fabricated via PLML and single-crystal substrates were conducted using a diamond indenter equipped in an SEM. B_4_C, B and newly fabricated TiC were used as hard and brittle SMPs. Not only a hard substrate, such as SiC, but also softer substrates, such as Si and Al, were used to compare the mechanical behaviors using an embedment process at submicrometer scale. In particular, the possibilities of nano-Brinell hardness measurement and local surface enforcement were explored by analyzing the particle embedment process.

## 2. Materials and Methods

### 2.1. Particle Fabrication

Brittle SMPs of B_4_C, B, and TiC were fabricated via PLML and used for mechanical tests. The fabrication procedures of B_4_C and B have been previously reported [5,6]. Briefly, raw B NPs (Sigma-Aldrich, 7440-42-8, nominal size < 100 nm, Tokyo, Japan) were dispersed in deionized water for B SMPs and in ethanol for B_4_C SMPs and then irradiated using a Nd:YAG laser (Continuum, Powerlite Precision 8000, pulse width: 7 ns, wavelength: 355 nm, pulse frequency: 10 Hz) with a fluence of 200 mJ pulse^−1^ cm^−2^ for 10 min at room temperature. During laser irradiation, a magnetic stirrer was used to stir the liquid for agitation to disperse the raw particles. For B_4_C SMP fabrication, unreacted B was dissolved in nitric acid and removed. Common byproducts of boric acid for both processes were dissolved in water and removed. For TiC SMPs, TiC NPs (Sigma-Aldrich, 636967, <200 nm, Tokyo, Japan) were used as raw particles. A 532 nm Nd:YAG laser was irradiated at 150 mJ pulse^−1^ cm^−2^ in ethanol for 10 min and toluene for 120 min. The particles in toluene required a longer ultrasonication time for pre-dispersion (60 min) compared with those in ethanol.

### 2.2. Characterization

The morphology of the obtained spherical SMPs following laser irradiation was observed using a field emission scanning electron microscope (FE-SEM; JEOL JSM-6500F, Akishima, Japan). The crystallinity of the obtained particles was analyzed from X-ray diffraction patterns (XRD; Rigaku SmartLab, Akishima, Japan). The composition profile within individual particles was analyzed via energy-dispersive X-ray analysis (EDX) equipped with an aberration-corrected scanning transmission electron microscope (STEM; FEI Titan Cubed G2 60–300, Tokyo, Japan). The crystallinities of individual particles were measured via the electron diffraction technique using a high-resolution TEM (HR-TEM; JEOL JEM-ARM1300, Akishima, Japan).

### 2.3. Observation of Fracture and Embedment Behavior of SMPs

An indentation device (Hysitron PI SEM PicoIndenter) installed in a SEM (JEOL JIB-4600 F) was utilized for compressive fracture and embedment tests of the SMPs. The indenter speed for all compression tests was fixed at 12.5 nm s^−1^. Further detailed experimental conditions and the typical arrangement of the particles, substrate, and indenter tip were the same as those described in a previous report [4]. The diamond indenter tip was flattened to a 1 μm square, using a focused ion beam installed on the SEM, for the facile operation and observation of the particles during mechanical tests. This helped to ensure good reproducibility and repeatability. The substrates used for the mechanical tests of the particles were single crystals of SiC, TiO_2_, and SrTiO_3_, and polycrystals of Al (A5052), which have to be sufficiently flat and electroconductive for SEM observation during indentation. The particles must be sparsely distributed on the substrate to avoid compression of multiple particles with the indenter tip during measurements.

## 3. Results and Discussion

### 3.1. Fabrication of TiC SMPs by PLML

**Figure 1:**
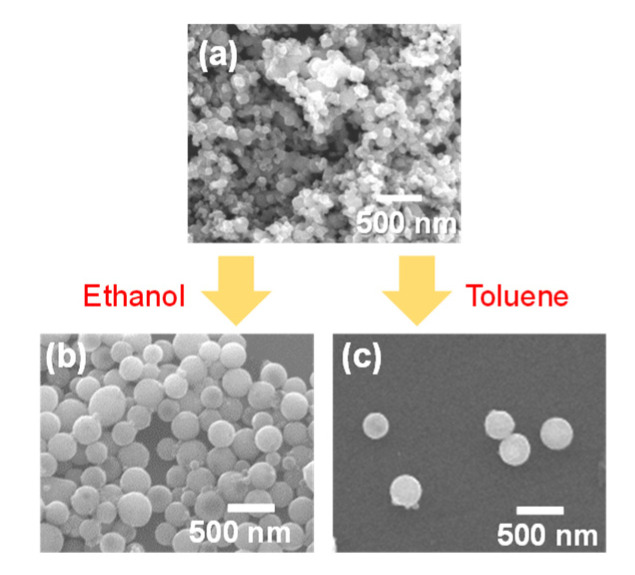
TiC SMP fabrication from TiC NPs via PLML. (**a**) SEM image of raw TiC NPs (<200 nm). SMPs obtained by 532 nm Nd:YAG laser irradiation at 150 mJ pulse ^−1^ cm^−2^ (**b**) in ethanol for 10 min and (**c**) in toluene for 120 min.

**Figure 2:**
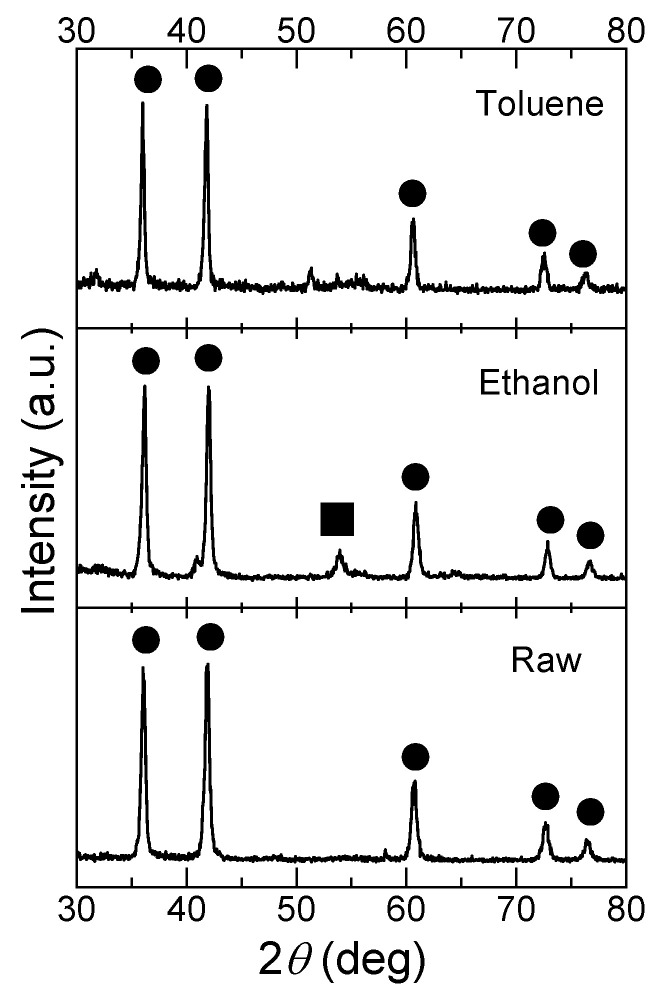
XRD patterns of raw TiC NPs and SMPs obtained via 532 nm Nd:YAG laser irradiation in ethanol and toluene. Circles indicate the TiC peaks, and the square indicates the TiO_2_ rutile phase.

**Figure 3:**
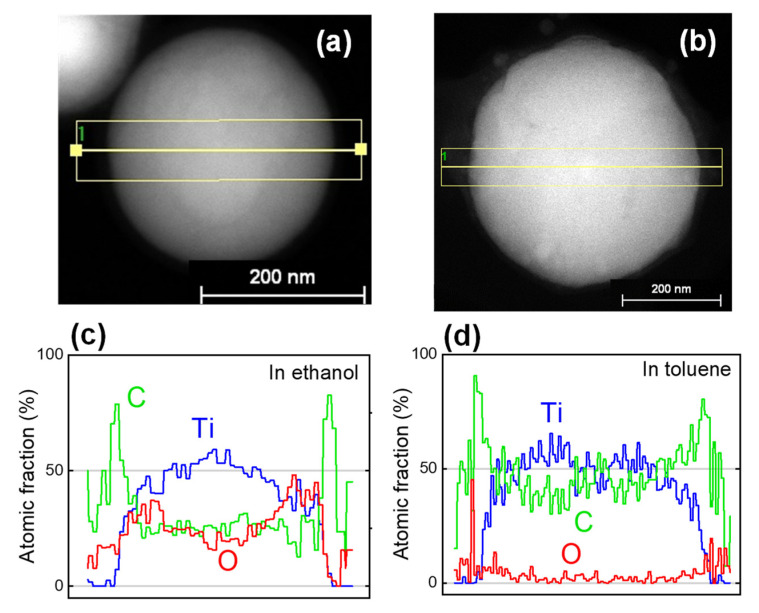
STEM images of TiC SMPs fabricated via PLML (**a**) in ethanol and (**b**) in toluene. The laser irradiation conditions were 532 nm Nd:YAG laser at 150 mJ pulse^−1^ cm^−2^ for 10 min in ethanol and 120 min in toluene. Compositional line scans of corresponding single particles obtained (**c**) in ethanol and (**d**) in toluene.

Figure 1 shows the SEM images of raw TiC NPs and particles fabricated via PLML in ethanol and toluene. Spherical particles were successfully fabricated in both solvents, forming SMPs with a similar size range from 300 to 500 nm. Figure 2 shows XRD patterns of the particles in Figure 1. Raw particles and particles obtained in toluene were pure TiC, whereas those prepared in ethanol contained TiO_2_ rutile phase, which was probably induced by reaction with oxygen in ethanol. Figure 3 shows the compositional line scans of particles prepared in ethanol and toluene obtained via aberration-corrected STEM. The data indicate that the particles fabricated in ethanol were oxidized at the surface and those in toluene were nearly oxygen-free. The electron diffraction technique using high-resolution TEM (not shown here) confirmed that the particles fabricated in toluene were single crystalline and those in ethanol were polycrystalline.

### 3.2. Fracture Strength of TiC SMPs Prepared by PLML

We previously reported fracture tests of B_4_C SMPs on SiC substrates using diamond tip indentation [4]. The fracture strength of the B_4_C SMPs was calculated to be 6–12 GPa, assuming tensile fracture at the center of a particle. This value was rather large compared with the typical bending strength by tensile fracture of a B_4_C sintered body (0.3–0.9 GPa) and was 18–38% of the ideal strength calculated by first-principles DFT. This high strength is due to the less defective nature of SMPs obtained via the PLML process.

**Figure 4:**
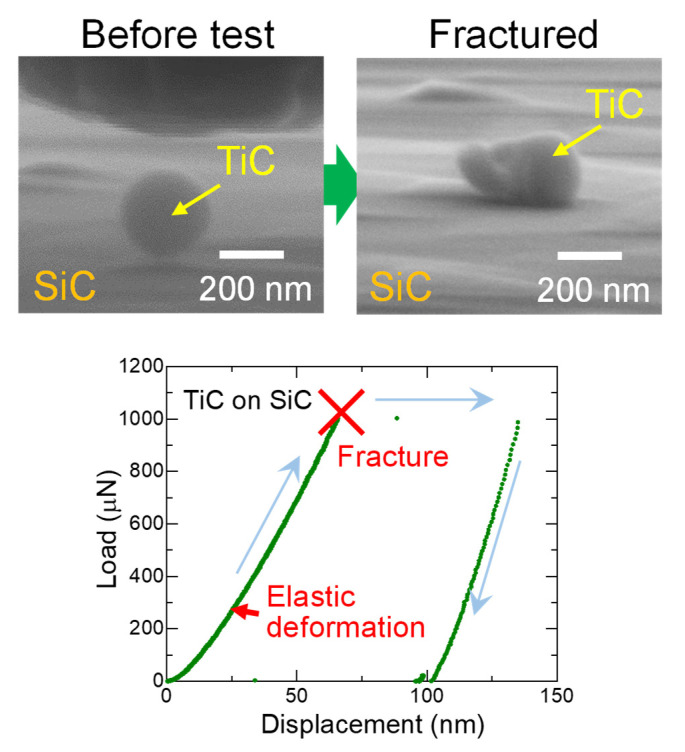
Typical fracture process of a TiC SMP and corresponding load–displacement curve during the compression test.

**Figure 5:**
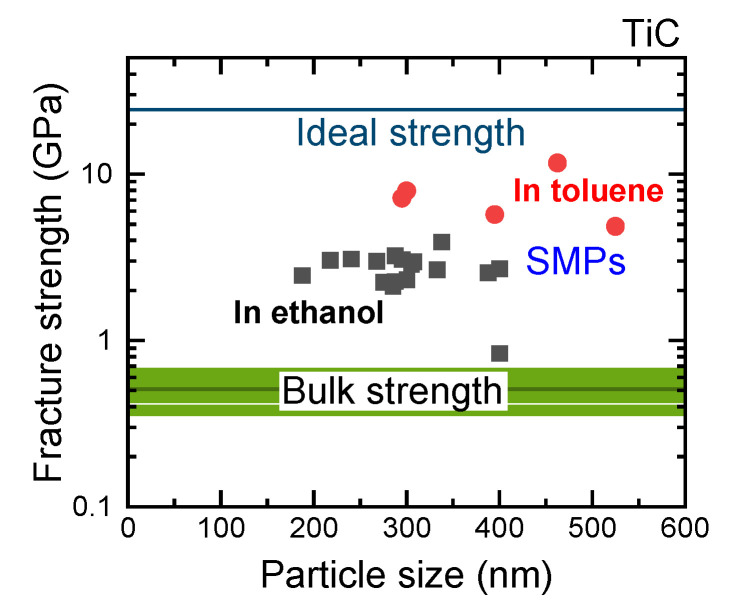
Particle size dependence of the fracture strength of TiC SMPs fabricated in ethanol (black squares) and toluene (red circles). The particles were placed on the SiC substrate and indented by a diamond tip. The calculated ideal tensile strengths and bulk strengths of large compact samples for TiC are also shown.

Figure 4 shows a typical fracture process of TiC SMPs on a SiC substrate using a diamond indenter with SEM images before and after the test. A clear jump of displacement induced by a fracture is observed in the load–displacement curve. Figure 5 shows the particle size dependence of fracture strength for TiC SMPs obtained using the Hiramatsu–Oka equation [18,19,20].

(1) $$S_{t}=2.8\frac{F}{\pi D^{2}}$$

where *S*_t_ (N m^−2^) denotes the fracture strength; *F* (N), the failure load of a spherical particle; and *D* (m), the particle diameter. The values indicate that the particles obtained in toluene (red circles, average fracture strength: 7.46 GPa) have higher fracture strength than those obtained in ethanol (black squares, average fracture strength: 2.67 GPa), although the average size obtained in toluene (396 nm) is larger than that in ethanol (301 nm), which is due to the difference in the permittivity of liquid, which controls the aggregation behavior of raw particles [6]. Larger particles generally have lower fracture strength due to the increased possibility of defect inclusion. However, our results indicate that the high fracture strength of particles obtained in toluene is caused by more perfect SMPs with less oxygen inclusion, as suggested in Figure 2 and Figure 3.

Figure 5 also shows a comparison of bulk bending strength [21,22,23], SMP fracture strength, and calculated ideal strength (24.4 GPa) of TiC [24]. The fracture strength of TiC SMPs fabricated via PLML ranged from 3% to 20% of the ideal strength when prepared in ethanol and from 20% to 50% when prepared in toluene. The previously reported fracture strengths of B_4_C and TiO_2_ SMPs obtained via PLML ranged from 18% to 38% and 10% to 40% of their respective ideal tensile strengths, which is similar to the TiC SMPs obtained in toluene. In relation to bulk fracture strength, TiC SPMs obtained in toluene were greater by one order of magnitude, as in the case of B_4_C [4].

### 3.3. Embedding Process of SMPs Obtained via PLML

**Figure 6:**
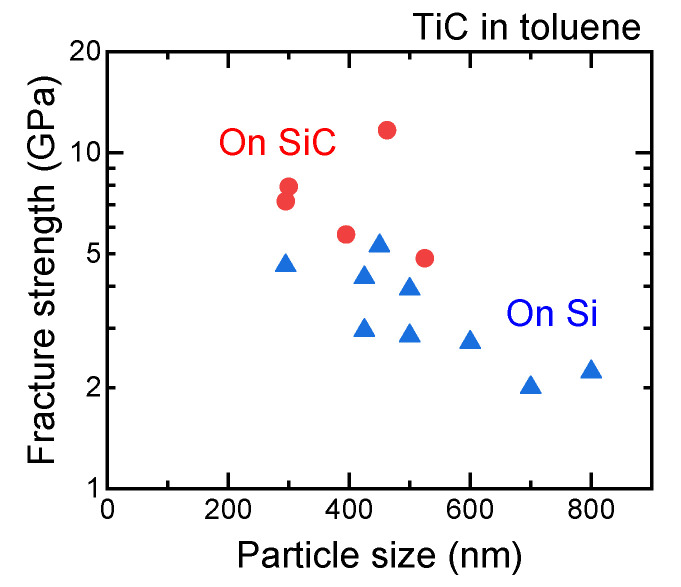
Particle size dependence of fracture strength of TiC SMPs fabricated in toluene. Fracture tests were conducted on SiC and Si substrates indented with a diamond tip.

The above data for the SMP fracture strength measurements were obtained using hard SiC substrates, and the particles were interlaid between the substrate and a diamond tip to be fractured. Si substrates can also be used for the indenter test of TiC, as shown in Figure 6. However, most fractured TiC SMPs on Si substrates were larger than 400 nm, which corresponds to the size range wherein phase-separated or polycrystalline SMPs tend to be formed in the PLML process [25]. Thus, the fracture strength gradually degraded with increasing particle size, as in the previous report [4]. In contrast, when smaller TiC SMPs were indented on the Si substrates, the particles were often embedded without appreciable shape change or after slight fracturing. This behavior may be observed on soft substrates and differs from the simple particle fracture process. Therefore, the embedding process of SMPs is systematically studied hereafter.

**Figure 7:**
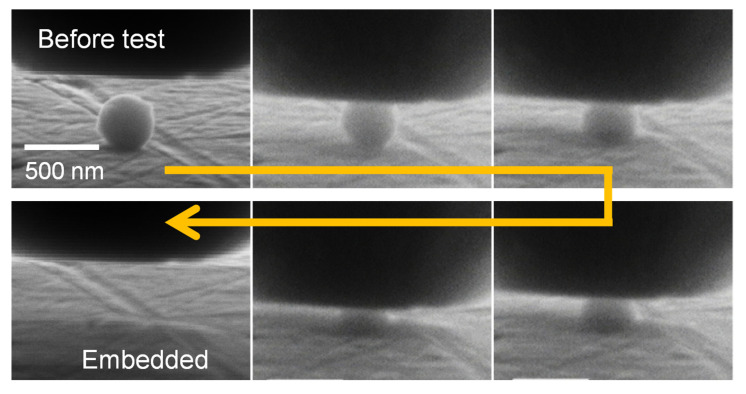
Embedment process of TiC SMPs on an Al substrate.

**Figure 8:**
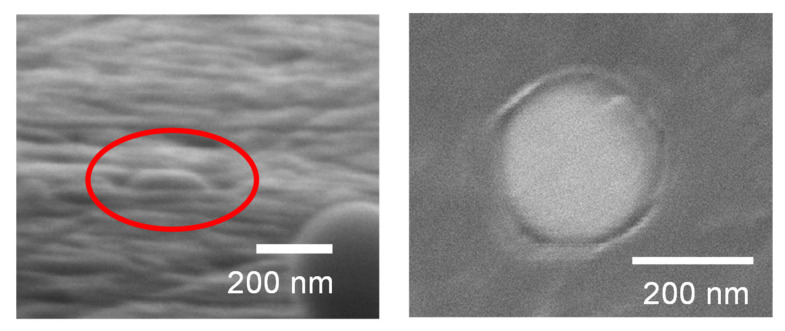
SEM images of the TiC-SMP embedded region on an Al substrate: left: oblique downward view; right: top view.

**Figure 9:**
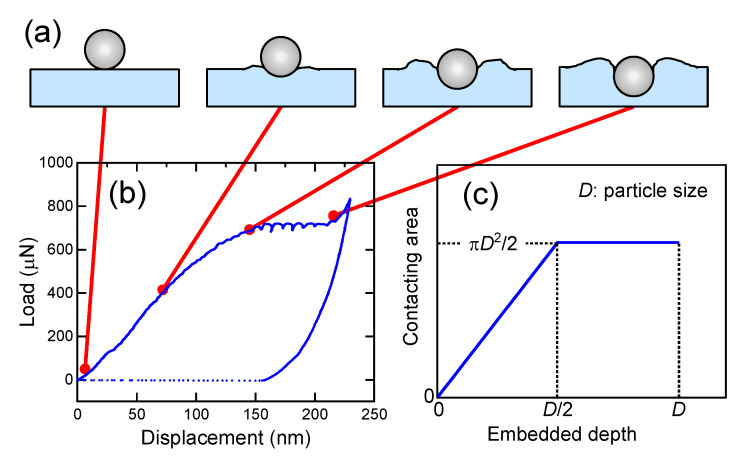
Embedding process of TiC SMPs on an Al substrate. (**a**) Schematic illustration of the particle embedment process. (**b**) Typical load–displacement curve of TiC SMPs on Al substrate. (**c**) Calculated relationship between the particle-surface contact area and embedded depth.

When soft Al substrates were used, the particles did not fracture but were gradually embedded, as shown in Figure 7. Figure 8 shows SEM images of the Al substrate after embedment of TiC SMPs, indicating complete embedment to the substrate level. Figure 9 shows a typical load–displacement curve wherein a particle is embedded and the corresponding schematic images illustrate how the particle is embedded. In contrast to the particle fracture in Figure 4, the particle is embedded with the load increment at the initial stage (Figure 9a) and then inserted into the substrate without a further load increase, exhibiting a plateau in the load–displacement curve (Figure 9b). This behavior is relevant to the contact area change with embedded depth, as shown in Figure 9c. When the particle is embedded by half, a further push is made only through the contacting hemisphere surface. Further pushing will cause the diamond tip to touch the substrate, resulting in a sharp rise in the load–displacement curve, as demonstrated at the right side of the curve in Figure 9b. Thus, the idealized relation between the contact area and embedded depth is schematically displayed in Figure 9c.

### 3.4. Hardness Estimation of Substrates via SMP Embedding Process

**Figure 10:**
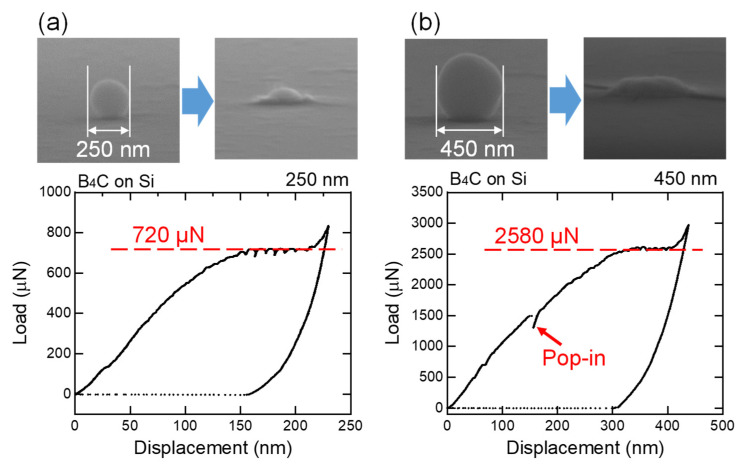
SEM images before and after the load application and load–displacement curves of the particle embedding process for (**a**) 250 and (**b**) 450 nm B_4_C particles in Si substrates.

Figure 10 shows the load–displacement curve of the particle embedding process for 250 and 450 nm B_4_C particles into Si single-crystalline substrates with SEM images before and after the load application. Nearly the entire particle was embedded after the load application, and only the top part of the particle can be observed after embedment. Plateau ranges in the load–displacement curves began when the displacement exceeded half of the particle size, suggesting the embedding process described in Figure 9c. The load at the plateau was larger for larger particles. For larger particles, a pop-in process, as in the case of 450 nm indented particles, is sometimes observed, indicating discontinuous embedment by crack formation of the particles [26].

With regard to the tip shape to push the substrate, the experimental arrangement in this study is similar to common Brinell hardness measurements using a macroscopic spherical tip, mostly made of a diamond of millimeter order in size and, recently, with micrometer range [27,28,29,30,31,32,33,34]. However, in our case, the particle indenter size was at the submicrometer scale, which is somewhat smaller than the conventional spherical indenter used for Brinell hardness measurements. When a large spherical tip was pushed onto the specimen, the Brinell hardness, *H*_B_, was calculated from the applied load, *F*; particle size, *D*; and measured indentation diameter, *d*, of the specimen in overhead view after unloading:

(2) $$H_{B}=\frac{2F}{\pi D\left(D-\sqrt{D^{2}-d^{2}}\right)}$$

In our experimental setup, a particle was continuously pushed during particle embedment. Thus, the displacement of the tip from the surface, *h*, can be adopted as the indentation depth, and the Brinell hardness can be calculated as follows:

(3) $$H_{B}=\frac{F}{\pi Dh}=\frac{F}{\left(\pi D^{2}\right)\left(h/D\right)}$$

**Figure 11:**
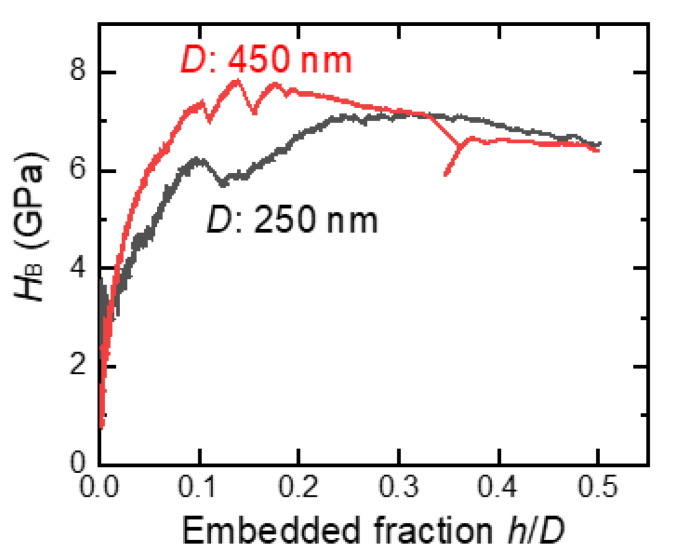
Brinell hardness variations calculated using Equation (3) from the initial stage of particle embedment data in Figure 10.

Figure 11 shows the Brinell hardness variations calculated using Equation (3) from the initial stage of particle embedment data in Figure 10. In the embedded fraction range of 0.25–0.50 (*d*/*D* range: 0.66–0.87), the calculated Brinell hardness values were close, irrespective of the particle size used to push the Si substrate.

The load value at the plateau range, *F*_p_, in Figure 10 can be easily determined since the embedded fraction, *h*/*D*, is 0.5 and, therefore, Equation (3) can be simplified as follows:

(4) $$F_{p}=\frac{\pi }{2}H_{B}D^{2}$$

**Figure 12:**
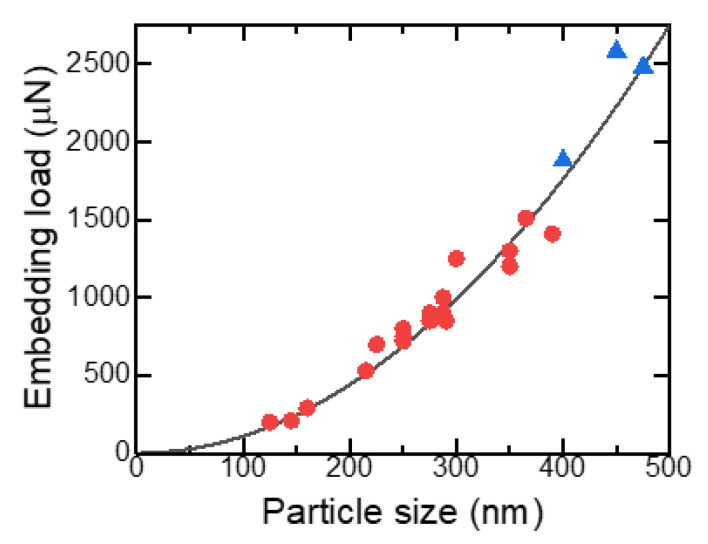
Particle size dependence of embedding load at the plateau region for B_4_C particles on Si substrate. Red circles and blue triangles are experimental data without and with pop-in behavior during indentation. The black curve is a fitted curve.

Figure 12 shows the embedding load at the plateau region in the load–displacement curve as a function of particle size for various B_4_C SMPs on a Si substrate. The solid black line is a curve fitted with a parabolic curve based on Equation (4) and is well fitted to the experimental data, indicated by red circles, without pop-in. The fit is also good for the data with pop-in behavior (blue triangles)—for particles larger than 450 nm. The Brinell hardness estimated from the fitting parameter in Figure 12 and Equation (4) was 7.01 GPa. Typical hardness values of Si reported were 11.1 (Vickers) [35] and 10.9 GPa (Berkovich) [36], obtained via microindentation tests, although the values were scattered from 7 to 20 GPa [37], depending on defect concentrations and distributions of the specimens and the difference in size, shape, and material of the indenter.

Nanoindentation techniques for hardness estimation have been developed to measure the mechanical properties of nano-objects. However, most nanoindentation devices use Vickers tips based on a pyramidal shape. Nanosized, sphere-shaped tips have seldom been utilized, probably owing to the unavailability of hard and small spherical particles. Spherical SMPs are a possible candidate for nano-Brinell tips to evaluate the hardness of nanosized objects.

### 3.5. Substrate Effects on the SMP Embedding Process

**Figure 13:**
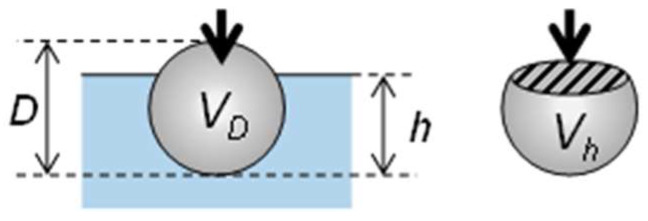
Schematic illustration of particle embedment.

Another possible application of hard and brittle SMPs is the site-selective surface post-enforcement of soft materials via particle embedment. For such enforcement to be sufficiently effective, particles should not be easily detached after embedment and, therefore, more than half of the particles have to be embedded. Particle embedment is completed when the indenter starts to push the substrate. The tip displacement on this occasion can be estimated from the right end of the load–displacement curve in Figure 9b. From the tip displacement at the embedment completion, *h*_c_; the particle size, *D*; embedded volume, *V*_h_; and particle volume, *V*_D_, (as presented in Figure 13), the embedded volume fractions can be calculated as follows:

(5) $$\frac{V_{h}}{V_{D}}=3{\left(\frac{h_{c}}{D}\right)}^{2}-2{\left(\frac{h_{c}}{D}\right)}^{3}$$

**Figure 14:**
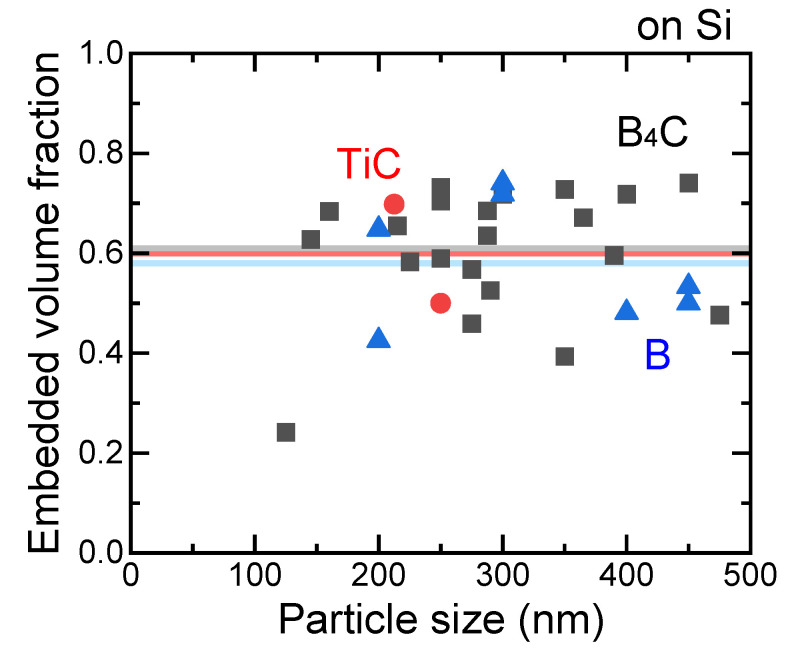
Particle size dependence of embedded volume fraction of TiC (red circles), B_4_C (black squares), and B (blue triangles) on Si substrates. The horizontal lines indicate the average values of the embedded volume fraction of particles with corresponding colors.

Figure 14 shows the embedded volume fraction as a function of particle size for various SMPs on Si substrates. Various hard brittle particles of TiC, B_4_C, and B were embedded in similar volume fractions (50–70%) into Si substrates, irrespective of the particle size. The average volume fractions, indicated by lines with corresponding colors, were nearly the same.

**Figure 15:**
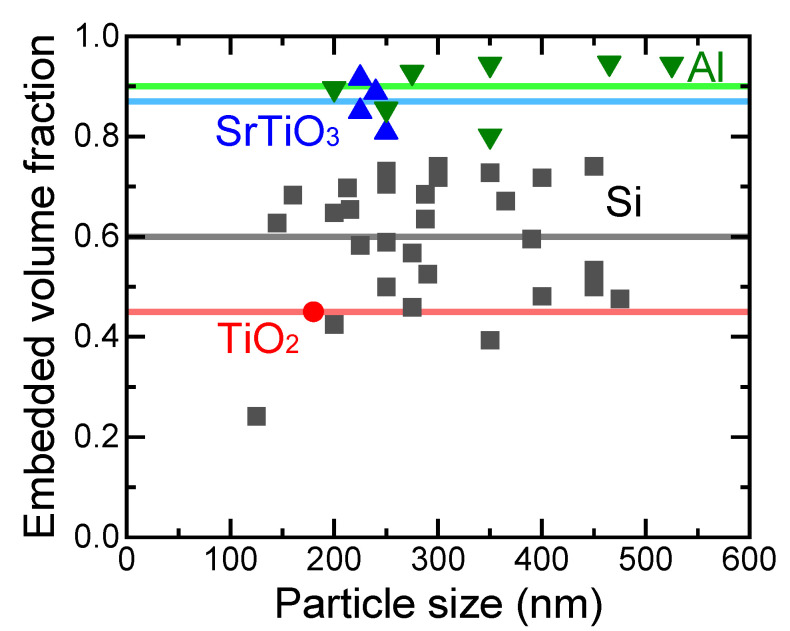
Particle size dependence of the embedded volume fraction for SMPs on various substrates of TiO_2_ (red circle), Si (black squares), SrTiO_3_ (blue triangles), and Al (green inverted triangles). The horizontal lines indicate the average values of the embedded volume fraction of particles with corresponding colors.

**Figure 16:**
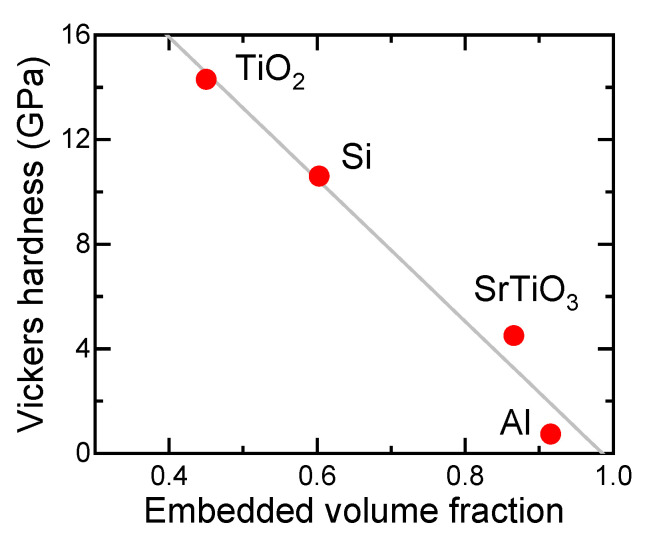
Relationship between Vickers hardness of substrates and average embedded volume fraction of SMPs.

Figure 15 shows the embedded volume fraction as a function of particle size for various SMPs on TiO_2_, Si, SrTiO_3_, and Al substrates. The embedded volume fractions do not have a clear size dependence in the case of Si substrates, though the embedded volume fraction increased with the decrease in Vickers hardness of the substrates, as presented in Figure 16. When more than half of the B_4_C, B, or TiC particles must be embedded, substrates with a Vickers hardness smaller than 12 GPa have to be used. Thus, site-selective surface enforcement is considered to be possible simply by spreading mechanically hard SPMs on a soft material surface and applying local force.

## 4. Conclusions

Spherical non-porous SMPs of hard and brittle ceramics, such as B_4_C, TiC, and B, were experimentally fabricated via the PLML process, which employs instantaneous pulsed laser heating of raw particles dispersed in a liquid. To explore and evaluate their potential for mechanical applications, these hard SMPs were compressed using a diamond tip on various substrates. The hard particles were fractured on hard substrates, such as SiC, and embedded into soft substrates, such as Si or Al. From the fracture process, particle strength can be estimated to be 5–12 GPa, which is larger by one order of magnitude than the bulk bending strength and 10–40% of the ideal strength of 24.4 GPa from the DFT calculations. From the embedment process, the submicrometer-scale Brinell hardness of 7.01 GPa of the Si substrate can be estimated by analyzing the load–displacement curves. The relationship of particle embedding depth and the hardness of substrates was elucidated from various combinations of SMPs and substrates.

## Data Availability

The data are included in the main text.
